# Integrative Analysis of Gene and Protein Expression Data Reveals Novel Clusters for Ovarian Cancer Prognosis

**DOI:** 10.3390/ijms27167144

**Published:** 2026-08-09

**Authors:** Jisup Kim, Seong Beom Cho

**Affiliations:** 1 Department of Pathology, Gil Medical Center, College of Medicine, Gachon University, Incheon 21565, Republic of Korea; 2 Department of Biomedical Informatics, Gil Medical Center, College of Medicine, Gachon University, Incheon 21565, Republic of Korea

**Keywords:** ovarian cancer, multi–omics integration, prognostic clustering, gene–protein co–expression, survival analysis

## Abstract

Ovarian cancer exhibits clinical heterogeneity; reliable prognostic stratification remains challenging despite extensive biomarker research. We performed an integrative analysis of transcriptomic and proteomic data to identify prognostically distinct molecular clusters in ovarian cancer. Using The Cancer Genome Atlas dataset, RNA sequencing and reverse–phase protein array data from 146 overlapping samples were analyzed. Genes (*n* = 150) and proteins (*n* = 67) showing nominal associations with overall survival (*p* < 0.05) were integrated; *k*–means clustering identified two patient clusters with highly significant differences in survival (*p* = 8.59 × 10^−15^). The *SAR2* and *ACTN4* genes and their corresponding protein expression levels showed high correlations among the significant genes and proteins (correlation coefficients > 0.80). Gene set enrichment analysis revealed that genes regulated by the PAX2 and BCL6 transcription factors were significantly enriched. The identified clusters were validated across six independent public transcriptomic datasets comprising 617 patients; consistent survival separation was observed between the patient clusters (*p* < 0.05). Clustering based on immunohistochemistry–derived expression profiles of nine proteins in an independent patient cohort demonstrated a significant difference in survival (*p* = 0.04). Integrative analysis of multi–omics data provided robust prognostic stratification in ovarian cancer while capturing the underlying molecular and biological features.

## 1. Introduction

Ovarian cancer is a malignant tumor of the ovary and represents the third most common cancer among malignancies of the female reproductive system [1]. Owing to the limited availability of effective screening tests, patients with ovarian cancer are often diagnosed at advanced disease stages [2]. The overall 5–year survival rate for ovarian cancer is approximately 50% and varies according to stage, demographic factors, and hormonal status [2]. Although several biomarkers have been proposed for predicting prognosis in patients with ovarian cancer, accurately distinguishing favorable and poor prognostic groups remains challenging. Identification of prognostic subgroups is therefore essential for enabling a more personalized approach to patient care. To date, only a limited number of biomarkers have been extensively investigated for ovarian cancer. Serum CA–125 is the most frequently studied biomarker and has been widely used for disease monitoring, early screening, and prediction of overall survival and chemotherapy response [3,4,5]. Serum human epididymis protein 4 is another well–established biomarker that outperforms CA–125, particularly for early detection [6,7]. Furthermore, combining CA–125 and human epididymis protein 4 in the risk of ovarian malignancy algorithm score has demonstrated improved performance compared with that of either marker alone [8]. In addition to these markers, several others—including osteopontin [9], mesothelin [10], kallikrein [11], and transthyretin [12]—have been investigated, supporting the need to expand biomarker development toward broader multi–marker strategies.

With the advent of high–throughput technologies, multiple genes have been explored for biomarker development in ovarian cancer. While mutations in individual genes, such as *BRCA1* and *BRCA2*, have predictive value for prognosis and treatment response [13,14], whole–genome sequencing studies have demonstrated that integrative patterns of somatic alterations across multiple genes—including single–nucleotide variants and copy number variations—can stratify patients with ovarian cancer into prognostically distinct subgroups [15,16,17]. Transcriptomic analyses have further enabled identification of gene expression signatures associated with prognosis. For example, transcriptomic profiling of 222 ovarian clear cell carcinomas reveals molecular subtypes linked to prognosis and therapeutic response [18,19]. More recently, cell type–specific expression patterns derived from single–cell RNA sequencing, such as S100A9^+^ tumor cells and HK2^+^ cancer–associated fibroblasts, have been used to identify aggressive subgroups of ovarian cancer [20]. In addition to transcriptomic data, proteomic analyses have contributed to the discovery of ovarian cancer biomarkers [21]. Proteomics–based studies have identified AAT (Alpha–1 Antitrypsin), NFKB (Nuclear Factor kappa–light–chain–enhancer of activated B cells), and PMVK (Phosphomevalonate Kinase) as prognostic biomarkers [22], while another investigation reports eight proteins with altered expression in ovarian cancer [23]. Collectively, proteomic analyses have revealed biomarkers associated with prognosis [22,24] as well as chemotherapy responsiveness [25].

In this research, we integrated transcriptomic and proteomic data to find novel prognostic biomarkers for ovarian cancer. Rather than relying on a single gene or omics platform, we combined multiple genes and proteins for the determination of patient clusters with different survival outcomes. Importantly, the prognostic clusters were further validated using independent transcriptomic datasets and a separate protein–based dataset.

## 2. Results

### 2.1. Preprocessing of Ovarian Cancer Multi–Omics Data

The multi–omics data of ovarian cancer from The Cancer Genomics Atlas (TCGA) project included 382 samples. RNA sequencing data (*n* = 256), normalized using the RNA–seq by Expectation and Maximization (RSEM) method, were used for analysis [26]. The protein expression data obtained using the reverse–phase protein array (RPPA) platform included only 146 samples [27]; therefore, RNA sequencing data from the same samples were used in the integrative analysis. Since 21% of the protein expression data were missing, the ‘impute’ R package (version 1.80.0) was applied for missing value estimation, and default parameters were used in the imputation process [28]. The *k*–nearest neighbor (KNN) imputation method was selected because it showed the best performance in previous studies and is one of the most widely used imputation methods in genomics. This widespread acceptance and historical performance formed the basis for using the KNN imputation method [29].

The TCGA ovarian cancer dataset also provided clinical information on disease–free survival and overall survival status. Follow–up status and overall survival duration were used as clinical endpoints. Of 587 patients, 236 survived until the last follow–up, with a mean follow–up duration of 38.75 months. The mean survival time of deceased patients was 52 months. Table S1 shows the clinical characteristics of patients in the survival and non–survival groups.

### 2.2. Identification of Clusters Having Significantly Different Survival Rates

After applying the Cox proportional hazards model to the gene and protein expression data, the significance of associations between expression profiles and overall survival was determined. The numbers of genes and proteins in the transcriptomic and proteomic datasets were 12,031 and 5874, respectively. When Bonferroni multiple testing correction was applied (*p* value < 4.16 × 10^−6^ for transcriptomics and <8.51 × 10^−6^ for proteomics), no significant associations were identified. Similar results were obtained using the Benjamini–Hochberg method with a more relaxed threshold (adjusted *p* value < 0.05).

**Figure 1:**
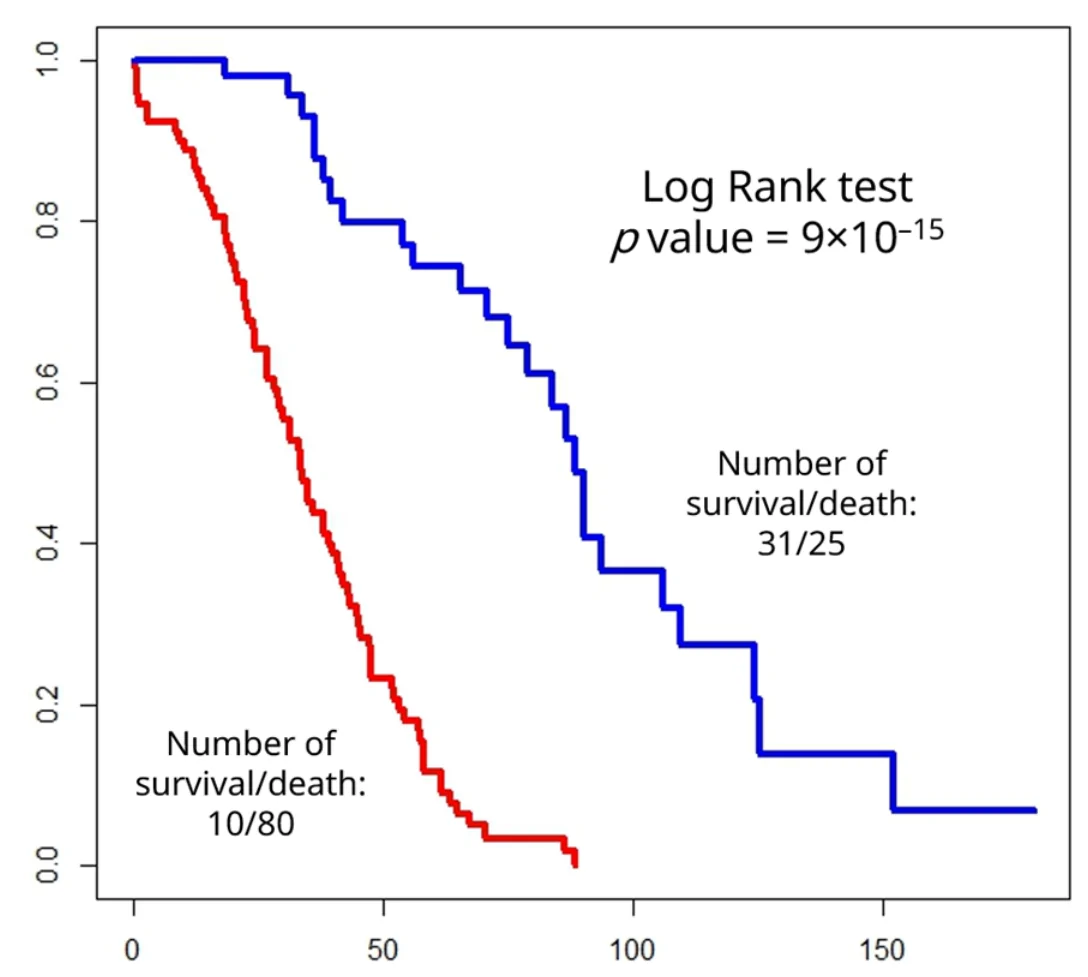
Kaplan–Meier curves of prognostic clusters determined by multi–omics data. The x–axis indicates patient survival in months, and values on the y–axis represent survival probabilities.

To identify patient clusters with differential survival rates, genes and proteins with nominal significance (*p* < 0.05) were selected. RNA sequencing and RPPA analyses identified 619 and 369 nominally significant genes and proteins, respectively (Table S2). As described in the Materials and Methods Section, expression profiles of these genes and proteins were integrated into a single dataset (number of variables = 988), and variables were ranked according to *p* values from the Cox proportional hazards model. The most significant genes or proteins were added sequentially until *k*–means clustering identified two clusters showing the greatest survival difference based on the log–rank test. When 217 variables were included, the log–rank test comparing the two clusters identified by the *k*–means algorithm yielded the most significant result (*p* = 8.59 × 10^−15^, Figure 1). These 217 variables comprised 150 genes and 67 proteins (Table S3). To assess whether this result occurred by random chance, a permutation test with 10,000 permutations in which cluster memberships were shuffled was performed. The result remained highly significant (*p* < 1.0 × 10^−5^, Figure S1), indicating that substantial molecular differences were embedded in the expression profiles of the favorable and unfavorable prognosis groups. Since we used data–driven optimization to identify the clusters in the discovery dataset, we performed a cluster stability analysis to rule out potential overfitting. To estimate cluster stability based on out–of–sample performance, we applied the clusterboot function from the fpc R package (version 2.2–14, *k* = 2, 1000 bootstrap iterations, *k*–means algorithm). Both clusters demonstrated high stability, yielding bootmean values above 0.85 (0.90 and 0.93), which indicates a low risk of overfitting.

### 2.3. Molecular Characteristics of Genes and Proteins Determining Differential Survival Rates

**Figure 2:**
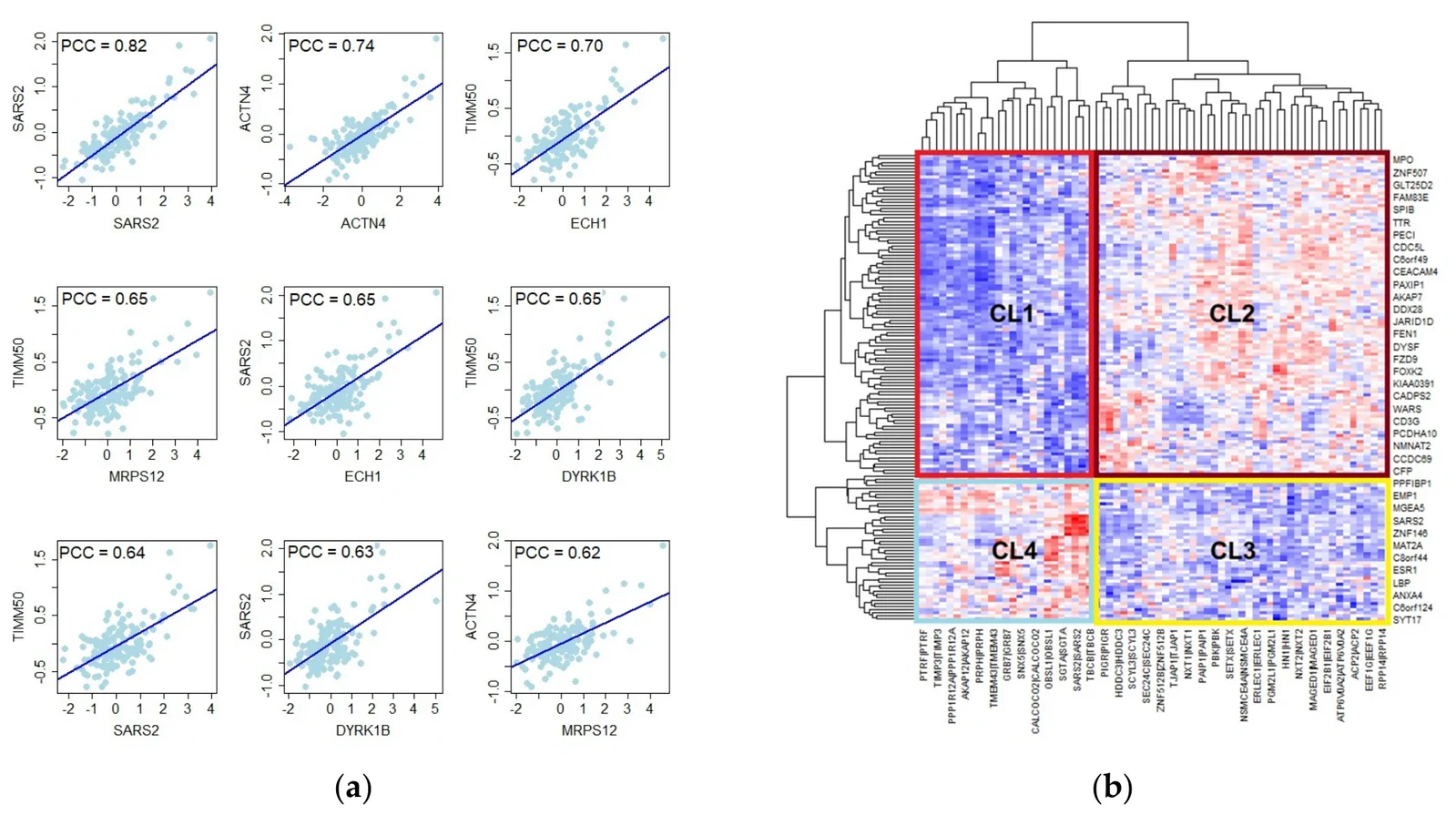
Co–expression analysis of gene and protein expression profiles nominally associated with patient survival. (**a**) Top nine gene–protein co–expression pairs showing significant associations after multiple testing correction. Gene names are shown on the x–axis, and protein names are shown on the y–axis. (**b**) Four clusters based on co–expression patterns between gene and protein expression profiles. *CL*, cluster; *PCC*, Pearson’s correlation coefficient.

After identifying clusters with differential prognoses, molecular characteristics and interactions were investigated. First, co–expression analysis identified 56 significant gene–protein pairs (Figure 2a and Table S4; Bonferroni–adjusted *p* value = 0.05/(150 × 67) = 4.98 × 10^−6^). Among these pairs, *SARS2* and *ACTN4* showed significant correlations with their corresponding proteins and exhibited the strongest and second strongest correlations, respectively (Pearson’s correlation coefficient [*PCC*] = 0.82 and 0.74; Figure 2a). Among the significant pairs, some proteins were associated with multiple significant gene expression correlations. For example, the SARS2 protein was significantly co–expressed with eight genes (*SARS2*, *ECH1*, *DYRK1B*, *MRPS12*, *ACTN4*, *FBXO17*, *LOC541469*, and *ZNF146*). The ACTN4 protein was significantly co–expressed with nine genes (*SARS2*, *PPFIBP1*, *ACTN4*, *DYRK1B*, *LOC541469*, *MRPS12*, *CLIP3*, *ZNF146*, and *ECH1*). Among these genes, *ECH1*, *MRPS12*, *DYRK1B*, and *LOC541469* were shared. In total, significant pairs included 27 genes and 17 proteins. After mapping proteins to their corresponding genes, the resulting gene list was subjected to gene set enrichment analysis using the gProfiler2 package [30]. The analysis revealed significant enrichment of genes associated with the Gene Ontology cellular component term “cell cortex” (*p* = 1.91 × 10^−2^; Table S5).

To capture broader co–expression patterns beyond individual gene–protein pairs, the full gene–protein correlation matrix was organized into co–expression modules using hierarchical clustering. First, the correlation matrix of genes and proteins (150 × 67) was constructed using all pairwise computations of *PCC*s. Subsequently, row– and column–wise hierarchical clustering was performed. When the number of clusters was set to two, four distinct clusters were identified (Figure 2b). Cluster 1 (*CL1*) contained 103 genes and 25 proteins, and FAM131A and GFAP showed the greatest absolute co–expression (*PCC* = −0.38, *p* = 2.76 × 10^−6^). Enrichment analysis revealed that genes associated with the gamma–delta T–cell receptor complex and BCL6 were significantly enriched (Table S6; *p* = 2.94 × 10^−2^ and 1.68 × 10^−2^, respectively). Cluster 2 (*CL2*) contained 103 genes and 42 proteins (Table S6). *FOXK2* gene expression and UBE2O protein expression showed the highest co–expression (*PCC* = 0.51, *p* = 7.05 × 10^−11^). Enrichment analysis of *CL2* showed that genes associated with gamma–delta T–cell receptor complex Gene Ontology terms were significantly enriched (*p* = 4.97 × 10^−2^; Table S6). Cluster 3 (*CL3*) contained 47 genes and 42 proteins, and the strongest co–expression was observed between *FOXK2* gene expression and UBE2O protein expression (*PCC* = −0.43, *p* = 5.88 × 10^−8^). Enrichment analysis revealed that genes related to microRNA hsa–mir–4708–3p were significantly enriched (*p* = 4.80 × 10^−2^). Cluster 4 (*CL4*) contained 47 genes and 25 proteins; interestingly, *SARS2* and *ACTN4* showed the most and second most significant co–expression with their corresponding proteins, respectively, consistent with the gene–protein co–expression analysis. Enrichment analysis of *CL4* showed that genes related to hsa–mir–3160–3p were significantly enriched (*p* = 4.51 × 10^−2^; Table S6).

### 2.4. Validation of Prognostic Clusters Using Public Gene Expression Data

To determine whether the clusters identified using the TCGA ovarian cancer dataset could be replicated in independent transcriptomic datasets, six public transcriptomic datasets were analyzed. Gene expression data and clinical follow–up information were obtained from the GEO database [31] (Table S7). Overall survival status and duration were used to define prognosis. Gene expression profiles from 719 patients were collected, and data from 617 patients with available survival status and duration were used for analysis. TCGA data were generated using an RNA sequencing platform, whereas the public datasets were generated using different microarray platforms; therefore, the transcripts represented differed across datasets. Using probe information provided in the GEO platform files, all probes were converted to gene symbols. When multiple probes mapped to a single gene symbol, probe–level expression values were averaged and used as the expression level for that gene symbol. Despite applying normalization procedures to each dataset (see Materials and Methods), principal component analysis revealed a clear batch effect (Figure S2). The ComBat function from the sva R package (version 1.9–1) was applied to correct this effect [32], and successful correction was confirmed (Figure S3). For a quantitative assessment of batch correction, we calculated the average silhouette width (ASW) using the silhouette function from the cluster R package (version 2.1.8.1). When analyzing principal component 1 (PC1) and PC2 from the raw validation dataset, the ASW was 0.74. This high value indicates a strong batch effect, occurring because the data points clustered predominantly by their respective batch groups. However, after applying batch correction, the ASW decreased to −0.11, indicating that the distribution of the data points was no longer correlated with the batch groups. Across datasets, 12,172 gene symbols overlapped, and expression data for these genes were used for validation.

**Figure 3:**
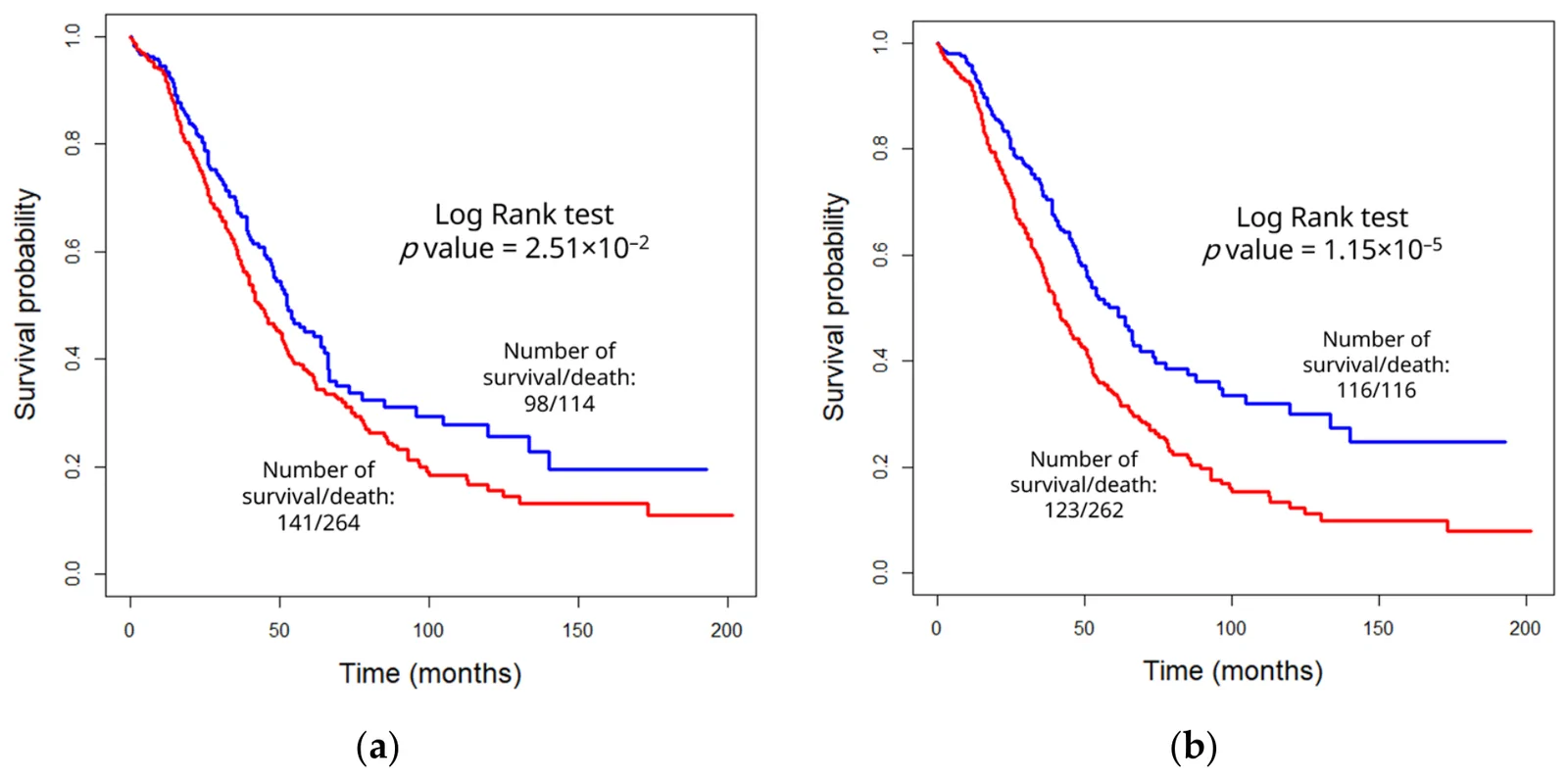
Clustering results using genes employed for prognostic cluster identification. (**a**) Kaplan–Meier curves of clusters determined using overlapping genes in the validation dataset (*n* = 119). (**b**) Kaplan–Meier curves of clusters based on expression patterns of 20 genes showing nominal significance in survival analyses of both The Cancer Genome Atlas and validation datasets.

Among the genes used to define prognostic clusters in the TCGA dataset, 119 transcripts overlapped with those present in the public transcriptomic datasets and were therefore used for independent validation. When *k*–means clustering was applied to TCGA patient gene expression profiles using these 119 genes, the resulting clusters showed significant differences in survival (*p* = 1.51 × 10^−11^; Figure S4). Applying the same procedure to the public datasets also yielded significant survival differences, although with reduced statistical significance (*p* = 2.51 × 10^−2^; Figure 3a). Because these 119 genes showed nominal significance in the TCGA survival analysis, the same genes were further evaluated in the validation dataset. Of the 119 genes, 20 showed nominal significance (*p* < 0.05) in the validation dataset. When expression profiles of these 20 genes were used for *k*–means clustering, the resulting clusters showed greater survival separation (*p* = 1.00 × 10^−5^; Figure 3b). These results were reproducible when the same analysis using the 20 genes was applied to the TCGA dataset (*p* = 7.00 × 10^−4^; Figure S5).

To rule out potential overfitting to the discovery dataset, we evaluated the reproducibility of the clusters using the centroid profiles from the *k*–means clustering of the discovery cohort. When applying the cluster centroids derived from the 119 genes, the resulting clusters within the validation dataset showed a significant survival difference (log–rank test, *p* = 0.03). Moreover, when this procedure was applied to the 20–gene expression matrix, the validation dataset clusters exhibited highly robust significance (*p* = 1.74 × 10^−5^). These findings demonstrate that the identified clusters possess clear prognostic predictability and substantial reproducibility.

### 2.5. Validation of Protein Markers That Are Used in the Determination of Differential Prognostic Clusters

**Figure 4:**
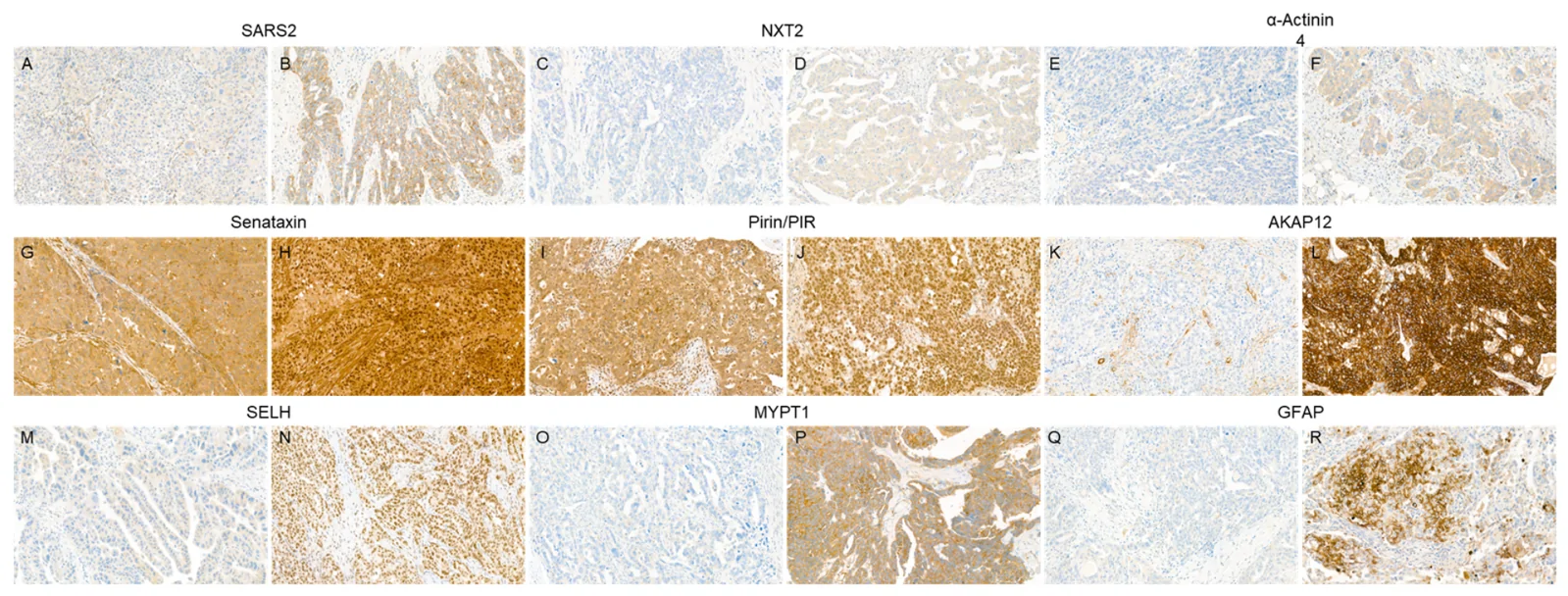
Representative immunohistochemical staining patterns of selected markers in high–grade serous carcinoma. (**A**,**B**) SARS2, (**C**,**D**) NXT2, (**E**,**F**) α–Actinin 4, (**G**,**H**) Senataxin, (**I**,**J**) Pirin, (**K**,**L**) AKAP12, (**M**,**N**) SELH, (**O**,**P**) MYPT1, (**Q**,**R**) GFAP. For each marker, the panel on the left shows lower expression (scores 0–1) and the panel on the right shows higher expression (scores 2–3). All images are shown at magnification ×400.

To validate the marker proteins used in the determination of the clusters, tissue expression of the proteins was assessed using immunohistochemistry (Figure 4). Proteins with the top 10 levels of significance in the survival analysis were identified, and nine proteins that were available for immunohistochemistry were selected. The original protein expression data were coded into four categories according to the magnitude of expression. These expression levels were discretized into two levels based on the survival time of the patients who provided the validation samples. For each protein, expression levels were assigned a value of zero or one according to a threshold level. For example, if a protein had four categories of expression (zero, one, two, and three) and the threshold was set to two, samples with values of zero and one were assigned a value of zero, whereas the remaining samples were assigned a value of one. Differences in survival time between the newly assigned zero and one groups were evaluated using the log–rank test. Likewise, test statistics were determined based on changes in the threshold. When a threshold produced the highest chi–square value in the log–rank test, that value was used as the discretization threshold for the corresponding protein.

**Figure 5:**
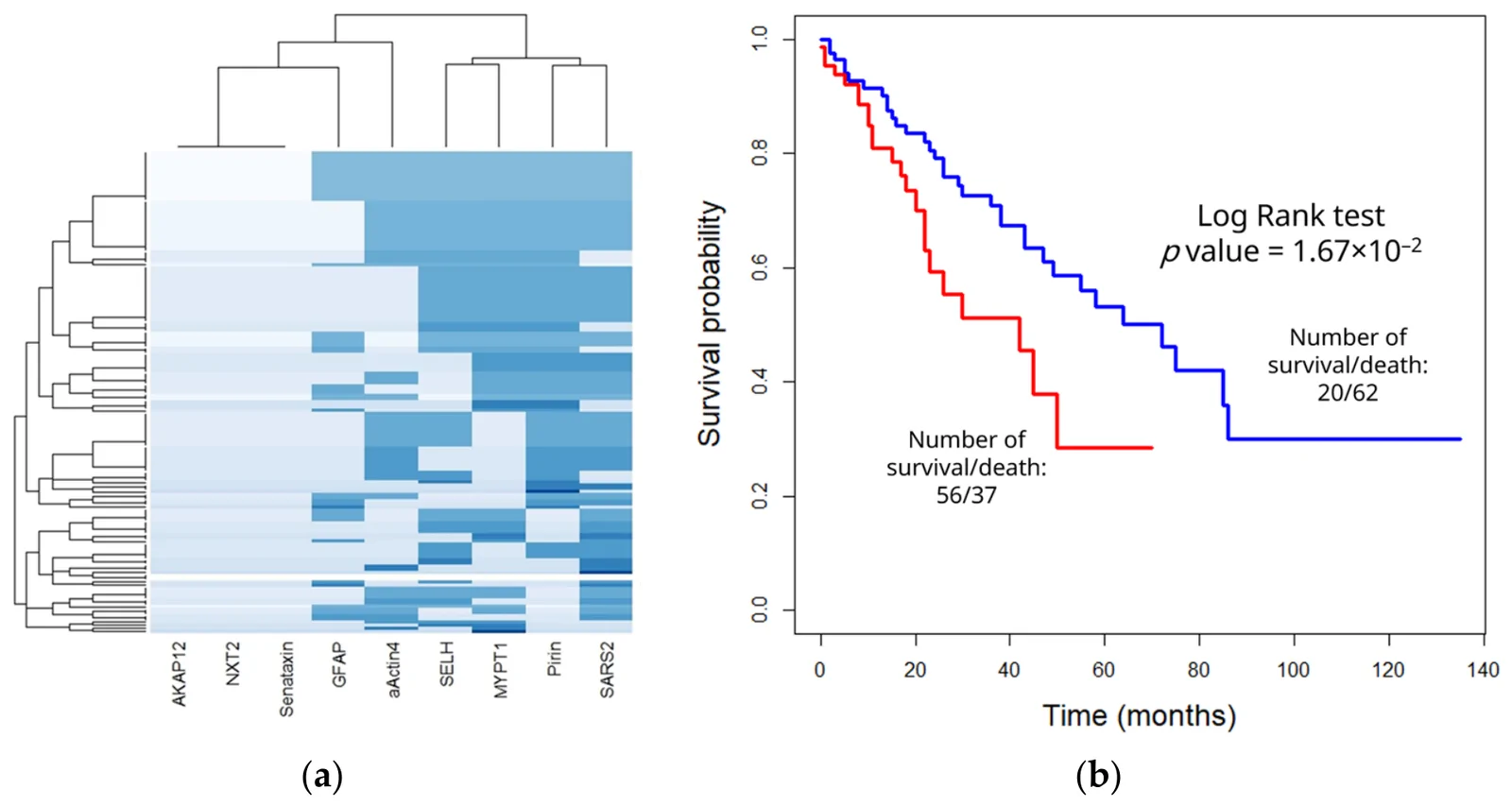
Validation results of nine proteins using clustering. (**a**) Heatmap of expression profiles of nine proteins showing distinct expression patterns across patients (y–axis). (**b**) Kaplan–Meier curves showing survival probabilities of two clusters determined by *k*–means clustering of discretized immunohistochemistry data.

After discretization, the log–rank test was applied to survival time, survival status of 155 patients, and protein expression. Of the nine proteins, only ACTN4 showed a significant difference (*p* = 0.03), whereas the remaining proteins did not show statistical significance (Table S8). The overall expression pattern of the data revealed two clusters (Figure 5a). When the *k*–means algorithm was applied to divide the discretized protein data into two clusters, the difference in survival duration between the clusters was significant (*p* = 0.04, Figure 5b).

## 3. Discussion

Using ovarian cancer multi–omics data, we identified novel patient clusters with distinct prognoses. Although individual genes or proteins achieved statistical significance, combining multiple variables yielded markedly bigger differences in survival probabilities. These cluster structures were successfully replicated across independent validation datasets.

In the cluster analysis, nominal *p* values were used strictly to rank variables rather than for formal hypothesis testing. Variables were then added sequentially based on this ranking to evaluate the statistical significance of survival distributions between the two clusters; thus, the initial nominal *p* values did not influence the final significance testing. For the discovery dataset, we initially set *k* = 2 to stratify patients into favorable and unfavorable prognostic groups. This choice was validated by silhouette analysis across the 217 variables for a range of *k* = 2 to 6, where *k* = 2 demonstrated the highest average silhouette width (0.08; see Supplementary Results). We therefore maintained the cluster number at *k* = 2.

The primary rationale for this multi–omics approach was to integrate complementary transcriptomic and proteomic profiles. Because ovarian cancer exhibits particularly low RNA–protein concordance, RNA expression alone cannot fully capture underlying biological processes and clinical phenotypes [33]. While generating multi–omics datasets remains challenging due to sampling constraints and high experimental costs, validation via single–omics platforms is highly feasible. Furthermore, selecting nominally significant genes or proteins for clustering increased their likelihood of replication in validation datasets. Supporting this strategy, gene expression data from platforms distinct from the TCGA dataset yielded concordant results.

The gene–protein co–expression markers and clusters identified here provide biological insights into the molecular processes driving prognostic heterogeneity in ovarian cancer (see Supplementary Results). For instance, *ACTN4* and *SARS2* demonstrated significant gene–protein co–expression, and both are implicated in ovarian cancer pathophysiology. High cytoplasmic ACTN4 expression correlates with serous histology, advanced FIGO stage, greater postoperative residual disease, and poor overall survival [34]. Additionally, *ACTN4* gene amplification (≥4 copies) is associated with platinum resistance, clear cell histology, and poor overall survival [35]. In contrast to previous studies spanning diverse histologic subtypes, this study focused specifically on high–grade serous carcinoma and confirmed worse overall survival in patients with high ACTN4 expression. Mechanistically, *ACTN4* encodes a non–muscle α–actinin isoform involved in actin cytoskeleton remodeling, cell motility, and invasiveness [34]. Unlike ACTN1, which stabilizes cell adhesion by interacting with β–integrin and α–catenin, ACTN4 localizes to the leading edge of motile cells to enhance migratory and metastatic potential [34].

Conversely, the prognostic relevance of *SARS2* has not been previously reported in ovarian cancer, though this mitochondria–related gene predicts poor prognosis in glioma and hepatocellular carcinoma [36,37]. Recent Mendelian randomization analyses indicate that *SARS2* expression promotes gastric cancer development by modulating CD8+ and CD4+ T–cell activity [38]. Notably, enrichment analysis of the co–expression pairs revealed a significant overrepresentation of genes regulated by the PAX2 transcription factor. While PAX2 is implicated in low–grade ovarian serous carcinoma development [39], its expression improves survival in animal models of high–grade serous ovarian cancer [40]. PAX2 promotes cancer cell growth by upregulating fatty acid metabolism [41], and its loss induces cancer stem cell markers [42].

Enrichment analysis of the gene–protein co–expression matrix provided regulatory insights into the prognostic clusters. *CL3* was enriched with *miR*–*4708*–*3p*–related genes. This microRNA downregulates *MDR1*—a gene driving chemotherapeutic drug resistance and poor prognosis in ovarian cancer [43,44]—and its higher expression predicts improved platinum response and longer treatment–free survival [45]. Thus, *CL3* likely reflects a molecular state associated with platinum responsiveness and favorable clinical outcomes. *CL2* was enriched for genes regulated by *miR*–*3160*–*3p*. While its role in ovarian cancer remains unclear, both *miR*–*3160*–*5p* and *miR*–*3160*–*3p* exhibit tumor–suppressor activity in prostate cancer [46], warranting further investigation in ovarian malignancies. *CL1* and *CL2* shared significant enrichment of the gamma–delta (γδ) T–cell receptor complex (Gene Ontology cellular component). The prognostic impact of γδ T cells is context–dependent: immunosuppressive, IL–17A–high subsets correlate with advanced FIGO stage, lymph node metastasis, and large tumor size [47], whereas functionally active, IFN–γ–high tumor–infiltrating subsets link to favorable survival [48]. *CL4* was characterized by strong *ACTN4* and *SARS2* gene–protein co–expression, consistently mirroring the aggressive tumor behavior and poor prognosis seen across multiple malignancies. *CL1* also showed unique overrepresentation of BCL6–regulated gene sets. BCL6 acts as a pro–oncogenic transcription factor in ovarian cancer; its overexpression correlates with advanced disease stages and poor prognosis by accelerating the G1–S cell cycle transition, proliferation, migration, and invasion [49]. Together, the concurrent enrichment of γδ T–cell signatures and the pro–oncogenic BCL6 transcriptional program suggests that *CL1* represents a highly aggressive molecular subtype.

In addition to analyzing the discovery dataset, we performed survival analysis on an independent immunohistochemistry (IHC) dataset incorporating tumor stage and neoadjuvant therapy status. To prevent overfitting from survival–based threshold selection, protein expression scores were binarized (set to 1 if >0, and 0 otherwise), and the dataset was partitioned into two clusters using *k*–means clustering. A Cox proportional hazards model incorporating stage and cluster membership showed no significant difference compared to a stage–only model (*p* = 0.67). However, incorporating neoadjuvant therapy status yielded marginal significance (*p* = 0.057). Given the sample size and number of proteins validated, these findings suggest that these clusters can contribute to prognostic predictions, particularly alongside neoadjuvant therapy data.

The proteins validated in these clusters play distinct roles in cancer progression and treatment resistance. For example, high levels of NXT2 are linked to poor outcomes in chromophobe kidney cancer [50], though its role in ovarian cancer was previously unstudied. SETX protects cancer cells by repairing DNA damage [51], and its response to cellular stress in ovarian cancer is tied to low survival and platinum chemotherapy resistance [52]. Similarly, Pirin acts as a stress sensor that drives inflammation, tumor growth, and shorter survival in liver and lung cancers [53,54].

Other proteins directly foster aggressive tumors. High AKAP12 expression correlates with poor survival and resistance to standard chemotherapy and anti–VEGF therapies [55,56,57] by promoting stem–like tumor properties and creating a hostile surrounding tissue environment [57]. SELH shields cancer cells from internal stress by boosting antioxidants, preserving genetic stability to help tumors survive and spread [58,59]. MYPT1 is activated by a tumor–promoting molecule in ovarian cancer abdominal fluid, driving cell migration, tissue invasion, immune evasion, and drug resistance [60]—aligning with data linking high MYPT1 to poor ovarian cancer outcomes [50]. Finally, GFAP, a structural protein tied to low survival in glioblastoma [61], is also expressed in reproductive system tumors [62] and correlates with worse outcomes in clear cell kidney cancer [50].

Because our primary objective was to identify prognostic markers using public transcriptomic datasets rather than to discover novel pathophysiological genes, experimental validation was outside the scope of this study. While definitive functional confirmation requires cell line experiments, we explored these functional roles using bioinformatic methods, including enrichment analysis and co–expression profiling. Our findings indicate that these genes likely contribute to ovarian cancer pathophysiology, warranting future experimental investigation.

Several limitations of the immunohistochemistry (IHC) validation cohort should be noted. First, using only a single 2–mm tumor core per patient creates potential sampling bias; while single cores generally match whole–section findings well for common ovarian cancer markers [63,64], they cannot fully capture the tumor’s internal spatial variation. Second, because a single pathologist scored all samples, we could not evaluate consistency between different observers. Finally, the cohort’s small size limited the statistical power to test individual protein markers (Table S9). However, our primary goal was to find overarching patient groups rather than isolated markers, and these broader groups still achieved statistical significance. Even so, future studies with hundreds of samples are needed to reliably validate individual proteins and confirm these patient clusters. Furthermore, because 217 variables were analyzed, directly building a predictive model would be underpowered even in a cohort of 1000 patients. Clinical translation will require dimensionality reduction methods to train models on realistic cohort sizes. Although profiling 217 variables is more expensive than testing a single marker, sequencing costs continue to decline steadily. Ultimately, these markers must be benchmarked against established clinical indices (e.g., tumor stage) and conventional biomarkers like CA–125 or HE4.

Finally, the study has several general limitations. Because it relied on existing, historical datasets, we lacked access to deeply detailed clinical information. Additionally, even though we used statistical methods to correct for variations between datasets, some hidden biases might remain because the data was generated under different lab conditions. There was also a mismatch in technology: our initial discovery dataset combined both protein and RNA sequencing data, but we could only validate these findings using older microarray data, meaning future studies should ideally use identical testing platforms. Lastly, because our sample sizes were relatively small, these findings cannot be easily generalized to all patients yet; moving this research into real–world clinical use will require validation in much larger, multi–center studies along with follow–up laboratory experiments.

In conclusion, we identified novel ovarian cancer patient clusters with distinct prognoses using multi–omics data. The functional implications of the constituent genes and proteins align closely with known ovarian cancer pathophysiology. These clusters were successfully validated using independent, publicly available transcriptomic and IHC datasets, highlighting their promise as robust future biomarkers. Ultimately, composite methods integrating both gene and protein expression metrics offer a powerful framework to enhance patient survival predictions.

## 4. Materials and Methods

### 4.1. TCGA Ovarian Cancer and Public Gene Expression Data

To identify novel prognostic clusters based on multi–omics data, the TCGA ovarian cancer dataset was downloaded from the cBioPortal website [65]. The multi–omics data from the TCGA ovarian cancer project included 583 samples. RNA sequencing data normalized using the RSEM method were used for analysis. RPPA protein expression data were available for 146 samples; therefore, the 146 overlapping samples with both transcriptomic and proteomic data were used for integrative analysis. Survival analyses were performed based on survival duration and overall survival status.

To validate the prognostic clusters, datasets containing ovarian cancer transcriptomic and survival data were obtained from the GEO database [31]. Using the keywords “ovarian cancer,” “human,” “GSE,” and “gene expression,” relevant datasets were retrieved. Only datasets containing more than 50 samples were included. Series matrix files containing survival and gene expression information were downloaded and parsed to extract relevant data. When transcriptomic data were not available in the series matrix files, raw data files were downloaded, and expression levels were determined using R packages specific to the corresponding platforms. For raw data generated using Affymetrix platforms, the justRMA function from the affy R package (version 1.86) was applied [66]. Quantile normalization was applied to all datasets, followed by sample–wise *z*–transformation, in which the difference between the original expression value and mean expression value of each sample was divided by the standard deviation of that sample, to reduce potential bias caused by scale differences across datasets.

Because the platforms differed among the public transcriptomic datasets, gene symbols were used to harmonize genes across different identifier systems. Gene symbols and other gene identifiers, including Entrez Gene IDs, were obtained from the corresponding GEO platform files. Using these files, gene identifiers from each dataset were mapped to gene symbols. When multiple probes or identifiers mapped to a single gene symbol, mean expression values were assigned as the expression level for that gene symbol.

### 4.2. Identification of Novel Prognostic Cluster Using Cluster Analysis of Multi–Omics Data

In the TCGA dataset, transcriptomic and proteomic data comprised different numbers of samples. Only samples with both gene and protein expression data were used for the analysis. First, only the gene and protein expression profiles were regressed on survival duration and survival status using a Cox proportional hazards model using the R program (version 4.5.1). Second, *p* values derived from the gene and protein expression profiles were merged into a single vector and sorted in ascending order. Third, using the list of genes and proteins ranked by *p* value, clusters were defined by incrementally adding variables. At each step, *k*–means clustering was applied to the expression matrix comprising the selected gene and protein expression profiles, and patients were divided into two clusters. Differences in survival probabilities between the two clusters were assessed using the log–rank test. This procedure was iterated (*n* − 1) times, where *n* represents the number of nominally significant genes (*p* < 0.05) identified in the survival analysis of the gene and protein expression profiles. Genes and proteins were selected at the step at which the log–rank test yielded the highest level of statistical significance.

### 4.3. Co–Expression and Survival Analysis with Clinical Data and Multi–Omics Data

To identify regulatory relations between genes and proteins used in the determination of prognostic clusters, co–expression analysis was performed using *PCC*.

(1) $$PCC=\frac{\sqrt{\sum_{i=1}^{n}\left(g_{i}-\bar{g})\times (p_{i}-\bar{p}\right)}}{\sqrt{\sum_{i=1}^{n}{\left(g_{i}-\bar{g}\right)}^{2}}\sqrt{\sum_{i=1}^{n}{\left(p_{i}-\bar{p}\right)}^{2}}}$$

In Equation (1) *g*_i_ and *p*_i_ indicate gene and protein expression levels, respectively. After computing the *PCC*, a statistical test was performed to estimate the *t*–statistic as follows:

(2) $$t=\frac{r\sqrt{n-2}}{\sqrt{1-r^{2}}}$$

In Equation (2), *n* represents the number of samples, and *r* denotes the *PCC* value. Bonferroni correction was applied for multiple testing of *p* values obtained from the co–expression analysis.

In addition to co–expression analysis, clusters of genes and proteins with similar co–expression patterns were identified using hierarchical clustering. First, the *PCC* matrix for genes and proteins was constructed by computing all pairwise PCCs between gene and protein expression profiles. Gene– and protein–wise hierarchical clustering was then performed, and two clusters were defined for each dimension. Finally, combinations of the two cluster memberships were used to identify groups with similar gene–protein co–expression patterns.

### 4.4. Validation of Protein Expressions Using Immunohistochemistry

Patients diagnosed with high–grade serous carcinoma after bilateral salpingo–oophorectomy at Gachon University Gil Medical Center were included. Only cases with formalin–fixed, paraffin–embedded tissue blocks available in the pathology archives were selected. Clinical data, including follow–up outcomes, were retrospectively collected from electronic medical records. Overall survival was defined as the interval from the date of surgery to death from any cause or last follow–up. This study was approved by the Institutional Review Board of Gachon University Gil Medical Center (approval number: GBIRB2023–314).

Tissue microarrays were constructed from available formalin–fixed, paraffin–embedded tissue blocks of high–grade serous carcinoma using one representative 2 mm core per patient. Sections of 4 µm thickness were cut from the tissue microarray blocks and subjected to immunohistochemistry. Immunohistochemistry staining was performed using an automated stainer (Bond Max; Leica Biosystems, Wetzlar, Germany). Heat–induced epitope retrieval was performed using EDTA–based buffers at pH 6.0 (ER1) or pH 9.0 (ER2), or Proteinase K treatment, as appropriate for each antibody. The following primary antibodies were used: anti–SARS2 (Abcam, Cambridge, UK; polyclonal; Cat. No. ab229227, 1:100, ER1); anti–NXT2 (Abcam; polyclonal; Cat. No. ab121797, 1:200, ER2); α–actinin 4 (Cell Signaling Technology, Danvers, MA, USA; D7U5A; Cat. No. 15145S, 1:200, ER2); senataxin (Novus Biologicals, Centennial, CO, USA; polyclonal; Cat. No. NBP1–94712, 1:200, ER2); pirin (Abcam; polyclonal; Cat. No. ab227280, 1:100, ER1); AKAP12 C–terminus (Abcam; polyclonal; Cat. No. ab204559, 1:500, ER1); SELH (Abcam; polyclonal; Cat. No. ab151023, 1:200, ER2); MYPT1 (Cell Signaling Technology; D6C1; Cat. No. 8574S, 1:100, ER1); and GFAP (Agilent Dako, Glostrup, Denmark; polyclonal; Cat. No. Z0334, 1:1500; Proteinase K).

Staining was evaluated by a board–certified pathologist using a BX53 light microscope (Olympus, Singapore), blinded to all clinicopathologic variables, treatment information, and survival outcomes. Subcellular localization of immunoreactivity was recorded according to the specific antibody target: cytoplasmic staining for SARS2, NXT2, α–actinin 4, AKAP12, MYPT1, and GFAP, and nuclear staining for senataxin, pirin, and SELH. Staining intensity was scored semi–quantitatively using a predefined four–tier scale: 0 (negative), 1 (weak), 2 (moderate), or 3 (strong).

## Data Availability

The original data presented in the study are openly available in GEO (https://www.ncbi.nlm.nih.gov/geo/, accessed on 28 October 2025) and cBioPortal (https://www.cbioportal.org/, accessed on 22 September 2025).

## Supplementary Materials

The following supporting information can be downloaded at: https://www.mdpi.com/article/10.3390/ijms27167144/s1.

## Abbreviations

The following abbreviations are used in this manuscript:

KNN	*k*–Nearest Neighbor
CL	Cluster
TCGA	The Cancer Genome Atlas
GEO	Gene Expression Omnibus
IHC	Immunohistochemistry
PCC	Pearson’s Correlation Coefficient
GO	Gene Ontology
PC	Principal Component
