# Supplementary material for: Integrative Analysis of Gene and Protein Expression Data Reveals Novel Clusters for Ovarian Cancer Prognosis
Source: Int J Mol Sci. 2026 Aug 9;27(16):7144. doi: 10.3390/ijms27167144 (PMC13513427; doi:10.3390/ijms27167144)
Supplement: Supplementary file 1 [file ijms-27-07144-s001.zip › Supplementary Results.pdf]

Supplementary Results

Determination of average silhouette width in the clustering of discovery dataset

To estimate the average silhouette width (ASW), we applied the silhouette function from the cluster R package (version 2.1.8.1) to the clusters generated by the *k*-means algorithm. The dataset consisted of 217 discovery variables, and *k* was evaluated from 2 to 6. The resulting ASW values for $k = 2$ through 6 were 0.08, 0.05, 0.03, 0.04, and 0.04, respectively.

Protein-protein interaction network analysis of significant gene-protein pairs

To biologically interpret the markers, we analyzed the genes and proteins that demonstrated significant mutual correlations. Mapping these genes to their corresponding proteins yielded 41 proteins for analysis. These proteins were queried in the STRING database, and Markov Clustering (MCL) was performed. Using the default MCL parameters implemented in the STRING database, the analysis identified six clusters that were tightly connected within the protein-protein interaction network. Furthermore, three clusters demonstrated potential connectivity (represented by dashed lines). Because proteins within the same cluster are often involved in the same biological processes, these results provide functional insights into the markers, highlighting them as potential targets for future functional studies.
